# Amino Acid-Based Polyphosphorodiamidates with Hydrolytically Labile Bonds for Degradation-Tuned Photopolymers

**DOI:** 10.1021/acsmacrolett.3c00173

**Published:** 2023-05-09

**Authors:** Stephan Haudum, Stefan Lenhart, Stefanie M. Müller, Disha Tupe, Christoph Naderer, Tilo Dehne, Michael Sittinger, Zoltan Major, Thomas Griesser, Oliver Brüggemann, Jaroslaw Jacak, Ian Teasdale

**Affiliations:** † Institute of Polymer Chemistry, Johannes Kepler University Linz, Altenberger Straße 69, 4040 Linz, Austria; ‡ Chair of Chemistry of Polymeric Materials, Montanuniversität Leoben, Otto-Glöckel-Strasse 2, A-8700 Leoben, Austria; § Institute of Polymer Product Engineering, Johannes Kepler University Linz, Altenberger Straße 69, 4040 Linz, Austria; ∥ School of Medical Engineering and Applied Social Science, University of Applied Sciences Upper Austria, 4020 Linz, Austria; ⊥ Tissue Engineering Laboratory, BIH Center of Regenerative Therapies, Department of Rheumatology and Clinical Immunology, Charité - Universitätsmedizin Berlin, Charitéplatz 1, 10117 Berlin, Germany

## Abstract

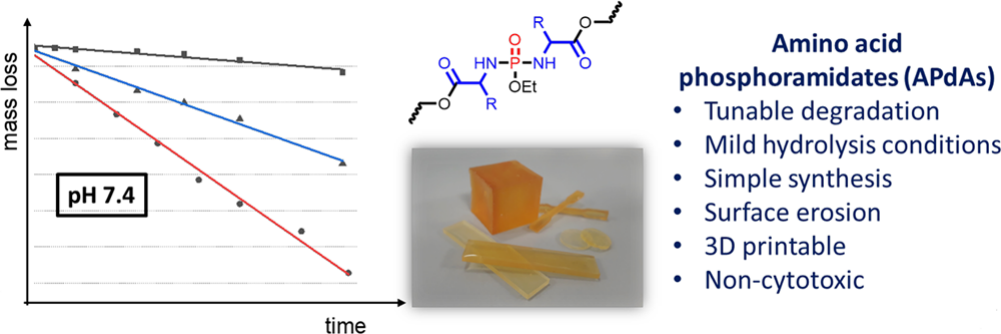

Photochemical additive manufacturing technologies can produce complex geometries in short production times and thus have considerable potential as a tool to fabricate medical devices such as individualized patient-specific implants, prosthetics and tissue engineering scaffolds. However, most photopolymer resins degrade only slowly under the mild conditions required for many biomedical applications. Herein we report a novel platform consisting of amino acid-based polyphosphorodiamidate (APdA) monomers with hydrolytically cleavable bonds. The substituent on the α-amino acid can be used as a handle for facile control of hydrolysis rates of the monomers into their endogenous components, namely phosphate and the corresponding amino acid. Furthermore, monomer hydrolysis is considerably accelerated at lower pH values. The monomers underwent thiol-yne photopolymerization and could be 3D structured via multiphoton lithography. Copolymerization with commonly used hydrophobic thiols demonstrates not only their ability to regulate the ambient degradation rate of thiol-yne polyester photopolymer resins, but also desirable surface erosion behavior. Such degradation profiles, in the appropriate time frames, in suitably mild conditions, combined with their low cytotoxicity and 3D printability, render these novel photomonomers of significant interest for a wide range of biomaterial applications.

Photochemistry is a powerful tool in polymer science, allowing exogenous control over network architecture and chemical functionality in a temporally and spatially controlled manner.^1^ In particular, additive manufacturing technologies (AMT), based on photopolymerization, have recently demonstrated their ability to produce high-resolution complex structures with tunable mechanical properties, e.g., via stereolithography (SLA), digital light processing (DLP), or 3D inkjet printing (3-DP).^2^ Such technologies enable, for example, the direct use of medical imaging to fabricate personalized medical devices such as individualized patient-specific implants, prosthetics, and tissue engineering scaffolds.^3^ However, there is limited availability of biodegradable photopolymer resins due to the necessity to balance degradation rates with the required functionality for photopolymerization. Established SLA methods, for example, rely heavily on acrylate and methacrylate chemistry, which is able to deliver the required fast-curing kinetics. However, such chemistry inherently produces high molecular weight aliphatic carbon backbone polymers,^2^ even when combined with biodegradable feedstocks.^3^

Alternatives have been developed in recent years, for example, photo-cross-linkable polypropylene fumarate, but degradation has only been evaluated with sodium hydroxide solutions (0.1 M NaOH),^4,5^ not at neutral and slightly acidic pH values more commonly encountered in biological applications. Other examples include the copolymerization of poly(ethylene glycol)-poly(lactic acid) (PLA) diacrylate macromonomers with thiols.^6,7^ Indeed thiol-based monomers with ester moieties integral in the network backbone have shown much promise in this field due to their superior mechanical performance and suitable curing kinetics.^8^ However, hydrophobic aliphatic esters also degrade very slowly in ambient conditions.^9,10^ Vinyl esters reportedly take many years at neutral conditions and stimulated in NaOH,^11^ and vinyl carbonates have been observed only to degrade at extreme pH values (1 M HCl and 1 M NaOH). A recently reported carbonate with a “fast” degradation was also only studied in 1 M NaOH.^12^ Hence, there is still a need in the field for degradable photopolymers with degradation properties that are matched to their application. This is paramount, for example, in the highly important field of tissue regeneration, whereby scaffolds must be carefully tuned to both the mechanical properties and growth rate of neotissue,^13^ and it remains a considerable challenge to design robust photopolymers with mild, ambient degradability.^14^

One method to accelerate the degradation rates of polyesters is the incorporation of cleavable moieties, as demonstrated by the incorporation of silyl ethers^15,16^ into acrylates or recently by Dove, who reported thiol–ene polymerizable polyorthoesters.^17^ Furthermore, Baudis recently prepared acetal-containing photopolymers that degrade rapidly at low pH values.^18^ This behavior is particularly suitable for bone regeneration scaffolds where pH values can drop relatively low.^20^ Incorporating phosphorus–nitrogen bonds into polymers can give cleavable linkages due to the susceptibility to hydrolysis.^19^ Wurm reported phosphorodiamidates and phosphoesters bearing alkene moieties that could be polymerized by thiolene chemistry.^20^ Subsequent oxidation gave water-soluble, linear, degradable polymers. The degradation rates were fast in acidic conditions, but slow under pH-neutral conditions.

Amino acids bonded to phosphates are known in biochemistry to hydrolyze readily; indeed, this hydrolysis reaction is the basis of many biochemical processes, for example, the transfer of phosphate from phosphocreatine to regenerate ATP from ADP.^21^ Herein we designed amino acid-based phosphorodiamidate (APdA) monomers bearing alkynyl moieties for subsequent thiol-yne photopolymerization. The good leaving group character of the amino acid was intended to increase the propensity to hydrolysis, with the α-substituent offering a handle to tune the hydrolysis rates through the steric hindrance of H_2_O. The alkyne-functionalized monomers (**1** Ala-APdA and **2** Gly-APdA) were prepared by the simple nucleophilic substitution of ethyldichlorophosphate with two equivalents of the respective amino acid alkynyl ester (Scheme 1). As a comparison, we also prepared the alkynyl phosphorodiamidate monomer **3** PdA, without an amino acid spacer via direct substitution of POCl_2_OEt with propargyl amine. The chemical structures and purity of the monomers were confirmed by ^1^H, ^13^C, ^31^P NMR spectroscopy and mass spectrometry (see SI-1–12).

**Scheme 1:**
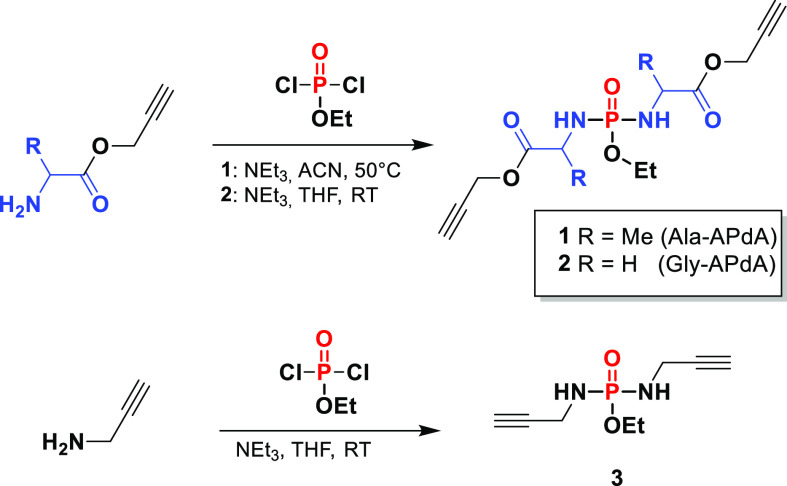
Monomer Synthesis Procedure Conditions for **1**: NEt_3_, ACN, argon, 50 °C, 14 h. Conditions for **2** and **3**: NEt_3_, THF, RT, argon, 14 h.

The hydrolytic stability of the monomers was then measured in D_2_O (1 M HEPES buffer, 37 °C) at pH 7.4 (Figure 1a). The ^31^P NMR resonance signal remained unchanged for the nonamino acid containing PdA monomer **3** throughout the observed period of 229 days (SI-13), suggesting considerable resistance toward hydrolysis for this compound. Meanwhile, the amino acid-containing monomers showed clear hydrolysis through cleavage of the P–NH bonds to eventually give phosphate, as evidenced by increasing peaks at 1.5 ppm (Figure 1d). The phosphate and amino acid degradation products were also confirmed by MS spectrometry (SI-14). Moreover, the rate of hydrolysis was observed to be faster for **2** Gly-APdA than for **1** Ala-APdA (Figure 1a). The slower hydrolysis is presumed to be due to the shielding effect of the methyl α-substituent, as this effect has also been observed for the hydrolysis of the structurally related polyphosphazenes^22,23^ and polyphosphonates.^24^ As it is reported that phosphorodiamidates are acid-labile,^20,25^ we also studied the hydrolysis at pH 3.0 (1 M citric acid/D_2_O). A considerable acceleration in phosphate formation was observed for all monomers at this pH (see Figure 1b and SI-15). A number of biomedical applications require polymer materials that degrade at lower pH, making this triggered degradation an interesting property.^18^

**Figure 1:**
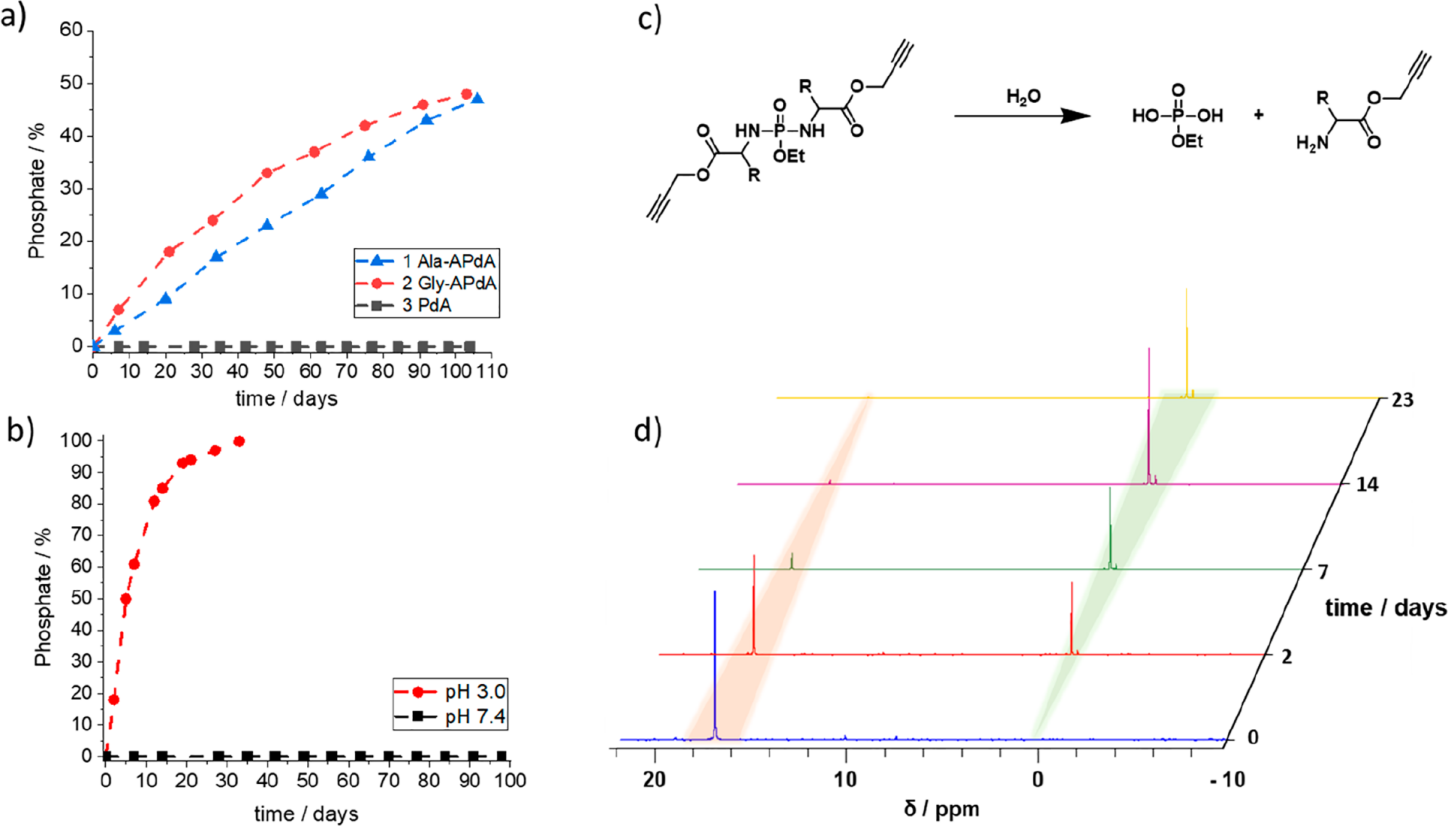
Hydrolysis was determined by phosphate production in ^31^P NMR spectroscopy. (a) Comparison of the monomer’s **1**–**3** hydrolysis at pH 7.4, 37 °C. (b) Comparison of the hydrolysis of **3** PdA at pH 3.0 and 7.4, 37 °C. (c) Hydrolysis reaction of the monomers. (d) Degradation of **2** Gly-APdA at pH 3.0, the monomer’s phosphorus signal at 17 ppm diminishes over time, while the new signal of the phosphate at 1.5 ppm arises. After 23 days, the monomer completely degraded to the corresponding phosphate.

The novel APdA monomers were then subject to photopolymerization with a stoichiometric ratio with the commonly used trithiol (1,1,1-tris(hydroxymethyl)-propane-tris(3-mercaptopropionate) (TMPMP). Similar systems have been widely studied with organic alkynes,^26,27^ acrylates,^28,29^ and vinyl esters^30^ for the preparation of photopolymers for a range of biomaterials applications. The monomer conversion (MC) was analyzed by RT-FTIR spectroscopy, in which the reaction was followed by a decrease in the alkyne band at 2130 cm^–1^ (Figure 2b, full spectrum SI-16). In addition, the depletion of the thiol moieties (2570 cm^–1^) and the formation of vinyl sulfide groups (2095 cm^–1^) as intermediates can be detected. Gly-APdA and Ala-APdA show slightly higher final MC than PdA (SI-17 and SI-18). The photokinetics were measured by photoDSC (Figure 2d and SI-19) with 5 wt% TPO-L as initiator. The three monomers all showed reasonable curing kinetics, with a *t*_max_ (time to reach maximum polymerization heat) between 8.6 and 9.7 s. The bulk mechanical properties of the resulting polymers were analyzed and displayed in Table 1 and depicted in SI-20 and -21. The values are comparable to similar thiol-yne-based polymers with TMPMP, but could be improved significantly in future generations through the use of alternative thiol comonomers.^30^

**Figure 2:**
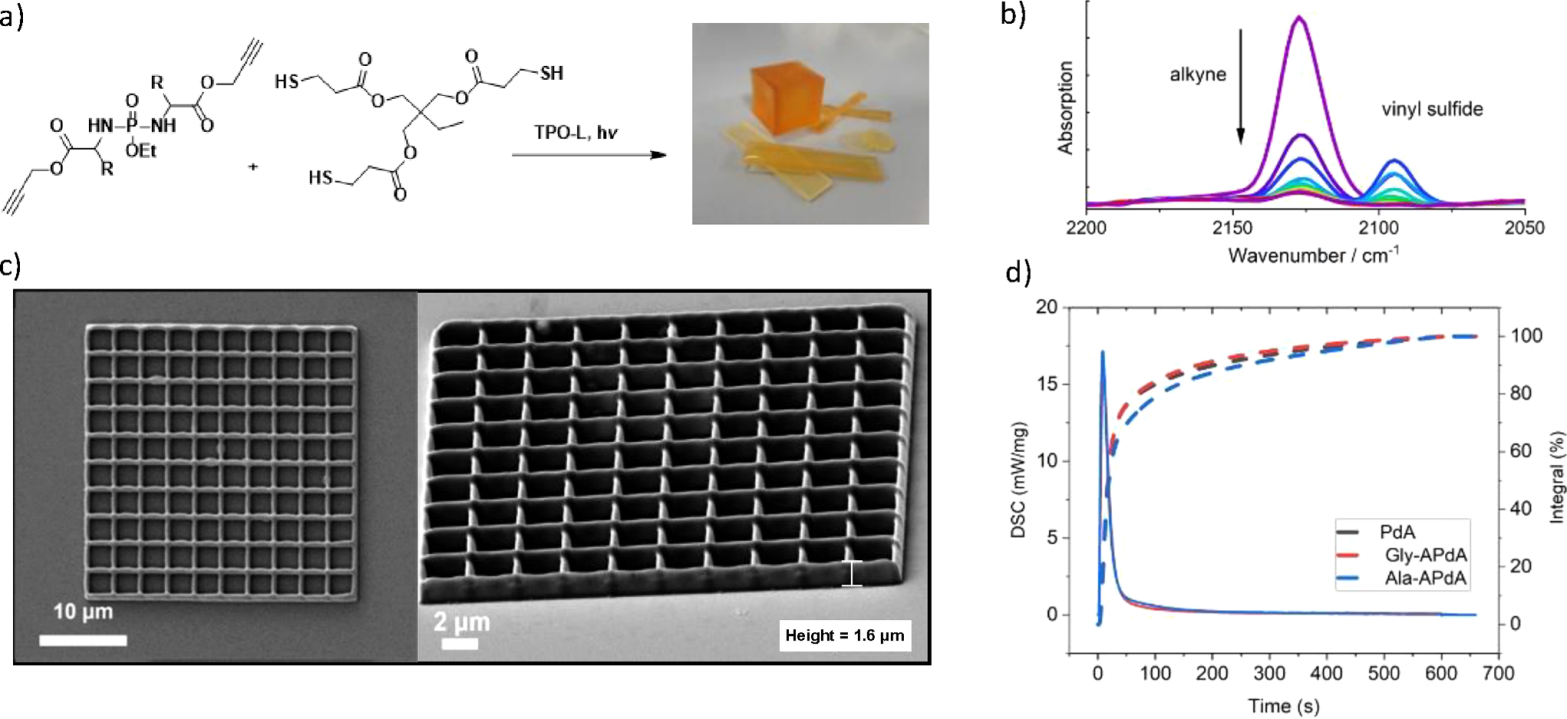
(a) Thiol-yne photopolymerization of APdA monomers, (b) RT-FTIR shows decrease in the alkyne band and the formation of vinyl sulfide groups as intermediates (full spectrum SI-15), (c) Gly-APdA 3D grid structure printed via multiphoton lithography (MPL). Left image top view, right image zoom on grid at 60° tilt. (d) The polymerization kinetics were measured by photo-DSC: The lines show *t*_max_, and the dashed lines show the percentage of the integral of the polymerization heat.

**Table 1:** Photochemical and Bulk Properties of the Thiol-yne Polymers

monomer	*t*_max_ (s)	MC (%)	η^a^ (mPa·s)	*E*^b^ (MPa)	σ^b^ (MPa)	*T*_g_ (°C)
PdA	8.97 ± 0.09	89.6 ± 2.0	135	15.2	4.6	26.3
Gly-APdA	9.74 ± 0.19	94.9 ± 0.38	321	18.8	5.6	29.4
Ala-APdA	8.56 ± 0.17	92.5 ± 0.71	257	10.6	3.4	32.6

a At 30 °C.

b 0.1 mm s^–1^, RT.

To demonstrate the applicability of the novel monomers for 3D printing, we prepared samples for multiphotolithography (MPL). Figure 2c shows a 30 × 30 × 1.6 μm^3^ grid fabricated with a custom-made lithography setup further described in the Supporting Information. The grid sidewalls consist of four partially overlapping excitations voxels (∼0.5 μm height for each) written on top of each other to enhance stability. MPL was performed with excitation power of 31 GW cm^–2^ and 7 μm s^–1^ writing speed (excitation wavelength: 515 nm fs-pulsed), illuminating each voxel twice.

We then studied the degradation behavior of the bulk polymers. Figure 3b shows the mass loss analysis of bulk materials under physiological conditions, pH 7.4 and 37 °C. A near linear decrease in mass and, hence, degradation of the sample was observed for the Ala-APdA (1) and Gly-APdA (2) containing samples. The degradation of the bulk material shows the same trend as the monomer degradation in solution in the order with the alkyne phosphorodiamidate **3** PdA < **1** Ala-APdA < **2** Gly-APdA. Indeed, the bulk material derived from the non-amino-acid-containing monomer **3** PdA shows only very minimal degradation in the measured time frame and conditions. This observation suggests that the potentially hydrolyzable P–N linkage of **3** PdA, as well as the ester bonds of the TMPMP are highly stable under these mild conditions in this polymer system. We observe a near-linear degradation of the prepared bulk polymers in the time frame and for the geometries studied, indicative of a surface erosion mechanism (Figure 3b). Surface erosion occurs when the rate of hydrolysis exceeds the rate diffusion of H_2_O into the bulk polymer.^32^ Diffusivity thus dictates the mechanism of polymer erosion for hydrophobic bulk materials and (as described by Andrianov and Sukhishvili^31^) is determined by factors such as crystallinity, swelling, and hydrophobicity^31^ The observation of surface erosion in our APdA-based polymers suggests that a low diffusion rate of H_2_O is rate-determining. This may be reasoned by the relatively low hydrophilicity introduced by the thioether bridges upon polymerization, which delays diffusion to the hydrolytically labile APdA groups embedded in the matrix (see contact angle measurements in SI-22). Furthermore, while the degradation of the pristine APdA monomers is strongly accelerated at pH 3.0, the pH value is observed to have little influence over the degradation process of the bulk material (see Figure 3a), further suggesting that the diffusion is rate-limiting and not the chemical reactivity. Surface erosion, generally expressed by a linear loss in mass over time, is a highly desirable yet rarely achieved property for biodegradable materials intended for use as biomaterials. Such behavior facilitates more uniform and thus predictable mechanical loss degradation and release of bioactives, if used for delivery purposes.^32^

**Figure 3:**
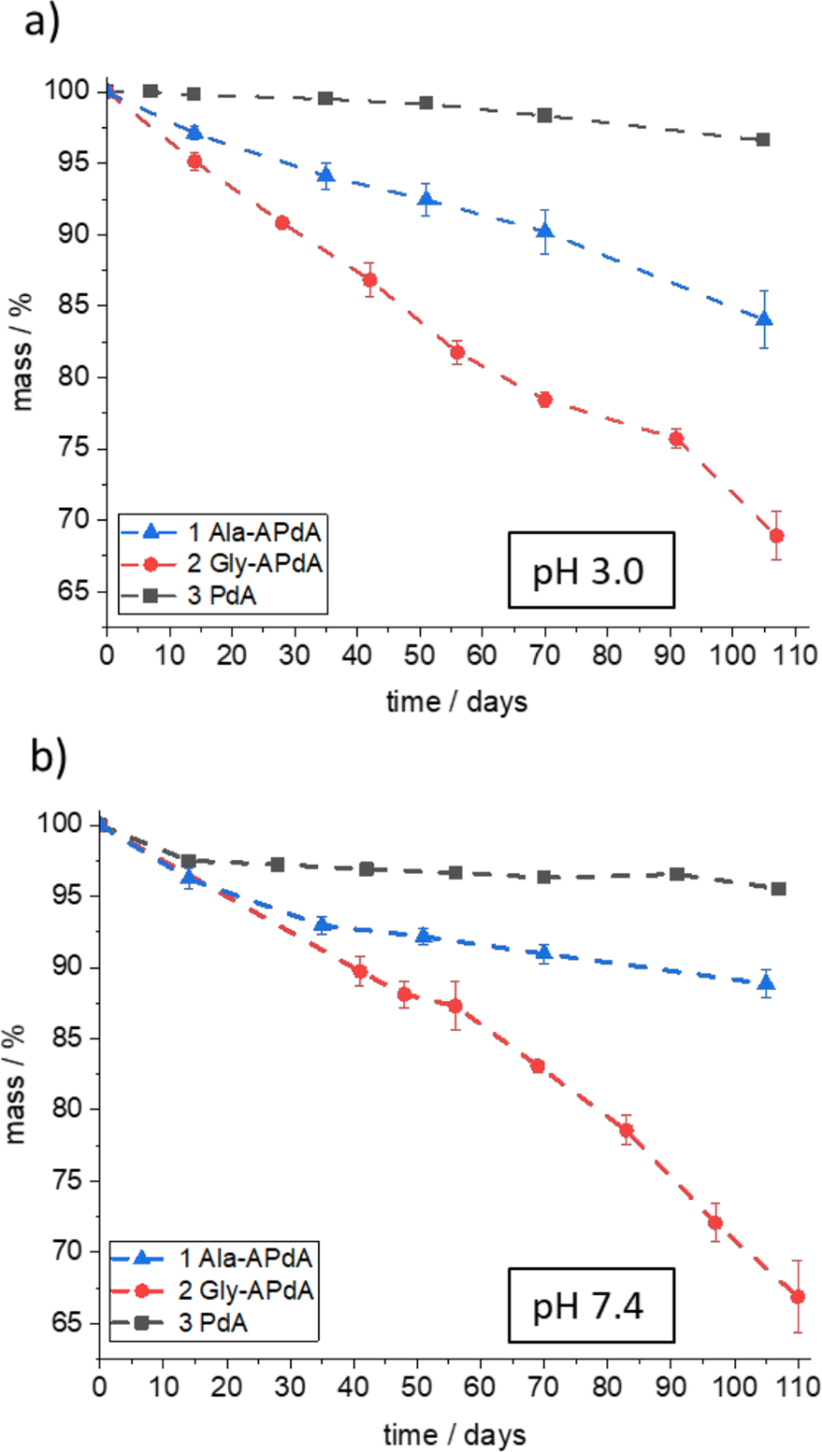
Diagram showing the mass loss tests (triplicates) of bulk materials at 37 °C in percent of *m*_0_ at pH 7.4 (b) and pH 3.0 (a) (error bars represent the standard deviation). While **3** PdA is stable at pH 7.4 and 3.0, monomers **2** and **3** show a gradual, near linear mass loss.

Cytotoxicity studies were carried out to provisionally assess the suitability of the novel materials as biological implants, adhering to existing standards for testing medical devices (DIN EN ISO 10993-5). Products leachable within 24 h under physiological conditions showed no relevant influence on the viability of the standard test cell line MC3T3-E1 (Figure 4a). Good surface adherence of the cells to bulk test specimens was observed, as well as cell-typical growth, with doubling times between 27 and 35 h (Figure 4b). Attachment of viable growing cells was detected by fluorescence microscopy on the surface of all material types (Figure 4c).

**Figure 4:**
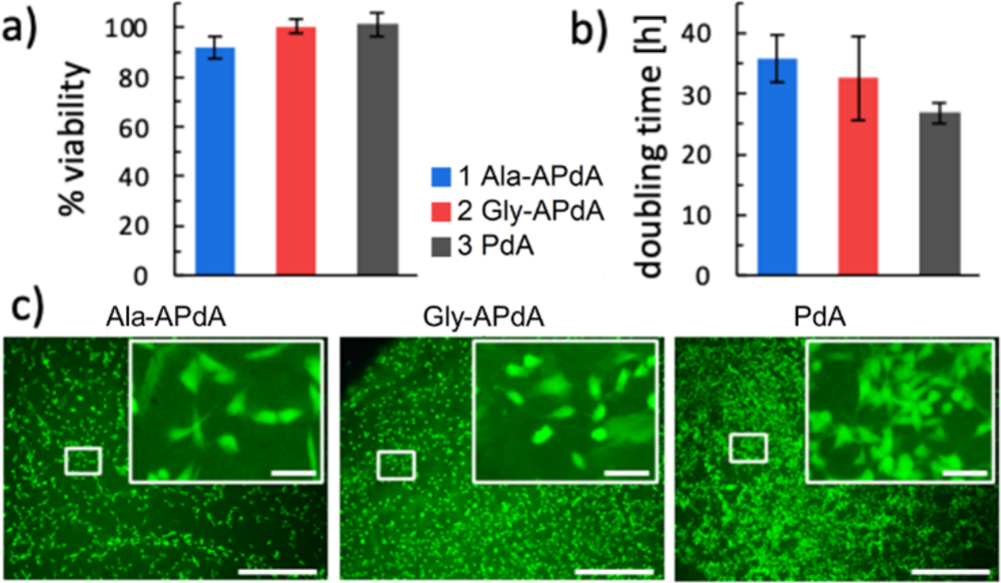
Cytotoxicity studies: (a) Diagram showing the viability of MC3T3-E1 cells maintained with eluates from the material. (b) Doubling time of adherent growing MC3T3 cells seeded on plane bulk materials. (c) Fluorescence microscopy images of viable MC3T3-E1 cells (green) grown for 72 h on the material surface. Bar = 500 μm, bar in inset = 25 μm.

In conclusion, we introduce a novel genre of hydrolytically cleavable phosphorodiamidate photomonomers with amino acid linkages. The amino acids are shown to cleave rapidly to the phosphate at pH 7.4, a process that is further accelerated at acidic pH. We study the photopolymerization of the APdA monomers in combination with known trifunctional thiols and demonstrate their 3D-MPL writing capability. The cured polymers were observed to have linear, pH-independent mass loss profiles, suggesting a surface erosion mechanism. The rate of hydrolysis could be tuned by choice of amino acid. This property, combined with the low cytotoxicity and cell-adherence of the materials and the endogenous nature of the main degradation products, makes these materials of significant interest for future development as degradable, 3D-printable biological scaffolds.

## Supporting Information Available

Experimental section, additional data for polymer characterization, and full NMR spectra (PDF)
