# Supplementary material for: Amino Acid-Based Polyphosphorodiamidates with Hydrolytically Labile Bonds for Degradation-Tuned Photopolymers
Source: ACS Macro Lett. 2023 May 9;12(6):673–8. doi: 10.1021/acsmacrolett.3c00173 (PMC10286305; doi:10.1021/acsmacrolett.3c00173)
Supplement: Supplementary file 1 — mz3c00173_si_001.pdf [file mz3c00173_si_001.pdf]

Supporting Information

Amino acid-based polyphosphorodiamidates with hydrolytically labile bonds for degradation-tuned photopolymers

Stephan Haudum[1], Stefan Lenhart[1], Stefanie M. Müller[2], Disha Tupe[3], Christoph Naderer[4], Tilo Dehne[5], Michael Sittinger[5], Zoltan Major[3], Thomas Griesser[2], Oliver Brüggemann[1], Jaroslaw Jacak[4] and Ian Teasdale[1]*

[1] Institute of Polymer Chemistry, Johannes Kepler University Linz, Altenberger Straße 69, 4040 Linz, Austria

[2] Chair of Chemistry of Polymeric Materials, Montanuniversität Leoben, Otto-Glöckel-Strasse 2, A-8700 Leoben, Austria

[3] Institute of Polymer Product Engineering, Johannes Kepler University Linz, Altenberger Straße 69, 4040 Linz, Austria

[4] School of Medical Engineering and Applied Social Science, University of Applied Sciences Upper Austria, 4020 Linz, Austria

[5] Tissue Engineering Laboratory, BIH Center of Regenerative Therapies, Department of Rheumatology & Clinical Immunology, Charité - Universitätsmedizin Berlin, Charitéplatz 1, 10117 Berlin, Germany

Supporting Information

Materials

If not stated otherwise all chemicals (Table SI 1) were used as received. All solvents and triethylamine were dried using 3 Å molecular sieves prior to use.

Table SI 1: Used chemicals, with purity, CAS and distributor.

**Chemical**	**Purity**	**CAS**	**Distributor**
Ethyl dichlorophosphate	97 %	1498-51-7	Thermo Scientific
Boc-Gly-OH	≥ 98 %	4530-20-5	Sigma Aldrich
Boc-Ala-OH	≥ 98 %	15761-38-3	Sigma Aldrich
Propargyl bromide	80 wt% in toluene	106-96-7	TCI
Potassium carbonate	≥ 99 %	584-08-7	Sigma Aldrich
Triethylamine	≥ 99 %	121-44-8	Sigma Aldrich
TPO-L	97 %	84434-11-7	BLDpharm
HEPES	≥ 99.5 %	7365-45-9	Carl Roth
Citric acid	≥ 99.5 %	77-92-9	Sigma Aldrich

Instrumentation

All NMR spectra (^{1}H, ^{13}C, ^{31}P) were recorded on a Bruker Avance II 300 MHz spectrometer.

DMA measurements were performed on a DMA Q800 from TA Instruments.

Photocuring of the samples was performed with a Rayonet RPR-200 Photochemical Reactor at 365 nm.

Contact Angle measurements were performed on a dataphysics OCA contact angle system at 21 °C.

Multi-photon lithography (MPL) was performed with a custom lithography system (Workshop of Photonics (WOP), Lithuania) utilizing a ultra-short pulsed laser at 515 nm (CARBIDE, 1 MHz repetition rate, >290 fs pulse duration, Light Conversion) and a 3-axis stage (AEROTECH Nanopositioner, USA) for sample movement, further described in ref[1] . An 63x magnification objective lens (63x, NA 1.25, Zeiss, Germany) was used for MPL. Formulation 1 (1.5g TMPMP, 0.8927g Gly-APdA) with 1%wt Irgacure 2959 (Sigma Aldrich, USA) as photoinitiator was used. Ethanol (Roth, Germany) was used for development.

Real-time FT-IR measurements were conducted on a Bruker (Billerica, USA) VERTEX 70 spectrometer with a A513 reflection unit. Multiple scans with a resolution of 4 cm^{-1} were acquired with a standby time of 2 seconds, resulting in a measurement interval of 5 seconds. For the sample preparation, TMPMP was added stoichiometrically to the corresponding monomers and 5 wt% of TPO-L was added as photo initiator. 1 µl of liquid sample was placed between two CaF_2 windows (10 mm diameter, 1 mm thickness) and placed on the gold mirror. Illumination was done with an Omnicure S1000 (EXFO Photonic Solutions, Canada) with an 8 cm gap between sample and light guide (75 mW cm^{-2} at the sample surface). The illumination was recorded for 200 scans (16.5 minutes). Atmospheric compensation was applied to suppress the signal of atmospheric carbon dioxide at 2350 ± 50 cm^{-1}. The monomer conversion (*MC*) was extracted from the recorded spectra after baseline correction as maximum height between 2150-2108 cm^{-1} for the alkyne signal. The final *MC* was calculated as a mean of the last 10 values recorded.

Photo-DSC experiments were performed on a NETZSCH (Selb, Germany) photo-DSC 204 F1 Phoenix. All measurements were conducted in open aluminium crucibles under nitrogen flow (20 ml min^{-1}) at 25 °C. For illumination an Omnicure S1500 (Lumen Dynamics, USA) was used at an intensity of 100 %, resulting in an irradiance of 292 mW cm^{-2} at the sample surface (sample quantity: 10 ± 1 mg resin). After an equilibration time of 4 min the samples were illuminated for 10 min twice, with an idle time of 1 min in between. For evaluation the second illumination step was subtracted from the first one and the time to peak maximum was evaluated for reaction kinetics.

Mechanical Testing

Uniaxial tensile tests are performed to determine the material characteristics of the material until it breaks, such as tensile strength, yield strength a, strain at break and other material properties. The test specimens were casted using PDMS moulds under laboratory conditions. The parameters were evaluated in accordance with the ISO 527 standard. The specimen size used was the ISO-527-5B type, which was the smallest size chosen. The thickness of the dog bone shaped casted specimen was 2 mm. The specimens were measured and tested at room temperature (23 °C). The uniaxial tension tests were performed with an MTS servo-hydraulic material testing system under isothermal conditions at a loading rate of 0.1 mm s^{-1} until specimen failure. The measurements were displacement controlled, and the force was recorded with a 250 N force cell. Prior to testing, the specimens were clamped with a clamping length of 17.3 mm, and to compensate for the clamping force that was applied, the specimens were elongated until the force was equal to zero. In the following, the initial length l_0 of the specimen was measured and the displacement-controlled testing was performed under isothermal condition. During the process, the force and displacement data were recorded for the creation of the stress-strain curve. $\sigma=F/A$ (equation 1 and $\varepsilon=(L-L_0)/ L0$ (equation 2 were used to calculate the tensile strength and strains:

$$\sigma = F/A \qquad \text{(equation 1)}$$

$$\varepsilon = (L-L_0)/L_0 \qquad \text{(equation 2)}$$

F …load prior to failure (N)

A …average cross-sectional area (mm^2)

L …actual displacement recorded (mm)

L_0 …clamping length when the force was equal to zero (mm)

The tensile strength is determined as the maximum stress that the material can sustain before it fractures. Young's modulus (*E*) is the slope of the linear elastic region of the engineering stress-strain curve. It is determined by dividing the stress by the corresponding strain within this linear region.

Biocompatibility test setup

Cell culture

The murine cell line MSC3T3-E1 (ACC 210, German collection of microorganism and cell culture GmbH) was used to test the biocompatibility of materials. According to the supplier’s recommendation, the

cells were propagated in cell culture medium (CCM) consisting of alpha minimum essential medium with ribo- and deoxyribonucleosides (α-MEM, GIBCO) supplemented with 10 % fetal calf serum, 4 mM L-alanyl-glutamine, 100 U mL^{-1} penicillin and 100 g mL^{-1} streptomycin (all from Merck). Cells were seeded at a density of 2-5 x10^3 cells cm^{-2}, and medium was changed after 72 h. At 90 % confluence, cells were detached with 0.05 % trypsin/EDTA and sub-cultured at least three times until use. For testing cell cytotoxicity of dissolvable products of the material MC3T3-E1 cells were seeded to 96-well plate with a density of 5,000 cells per well and maintained under standard cell culture conditions (37 °C, 5 % CO_2, 99 % humidity).

Cytotoxicity study

For testing cytotoxicity of dissolvable products, eluates of each material type were prepared. The material specimen (disc of 12 mm diameter, 2 mm height) was placed in a 24-well completely immersed with 1 mL CCM (described above) and incubated for 24 h under a continuous rock shaking in a cell culture incubator (37 °C, 5 % CO_2, 99 % humidity). Controls were generated by incubating CCM without a sample. 100 µL of the prepared eluate was added to a 96-well that was seeded for 24 h before. After 24 h incubation under cell culture conditions, the eluate was removed, and cells were washed with phosphate-buffered saline (PBS, Merck). Metabolic activity was determined with CCM supplemented with 10 % (v/v) CCK-8 reagent (Merck). After 2 h, absorbance at 450 nm and 650 nm was recorded with a spectrophotometer (Synergy HT, Biotek instruments). The absorbance difference (450 nm - 650 nm) corrected by a blank was related to the controls and presented as percentage viability. At least 5 samples per material type were tested.

Adhesion and growth

Material test specimens (discs of 12 mm diameter, 2 mm height) were placed in 24-wells. 0.5 mL CCM was added containing 25,000 MC3T3-E1 cells and incubated at 37 °C, 5 % CO_2, 99 % humidity. After 24 hours CCM was removed, the cell-laden material was washed with PBS, and transferred to a new 24-well. Fresh CCM (0.5 mL) was added and maintained under physiological conditions for additional 48 hours. Metabolic activity of the cells was determined at 24 h and 72 h post-seeding. After washing with PBS, 500 µL CCM containing 10 % (v/v) CCK-8 reagent were added and incubated for 2 h (37 °C, 5 % CO_2, 99 % humidity). Non-cell-seeded material samples incubated served as blank controls. Supernatant was transferred to 96-well-plate (100 µL each, n=5) and absorbance was measured at 450 nm and 650 nm with a spectrophotometer (Synergy HT, Biotek instruments). The absorbance difference (450 nm - 650 nm) corrected by the blank control was used to calculate the doubling time (t_D) as a measure for cell growth using the following formula

$$t_D = \ln 2\ (t-t_0)/\ \ln(A/A_0)$$

with $t-t_0$ = 48 h, A_0 as corrected absorbance measured at 24 h, A as corrected absorbance measured at 72 h. At least 4 test specimens were tested per material type.

The direct detection of adhered and proliferated cells was performed by fluorescence microscopy. Cell-seeded materials at 72 h after cell seeding (as described above) were washed with PBS and stained with 3 µg mL^{-1} fluorescein diacetate (Merck, dissolved in PBS) for 15 min). For microscopy, an Olympus CKX41 microscope combined with a reflected fluorescence microscopy system was used (Olympus, Hamburg, Germany). The staining results were photo-documented using the ProgRes® speed XT core5 camera and ProgRes® CapturePro 2.10 software (both Jenoptik, Jena, Germany). At least 4 specimen per material type were tested.

Experimental procedures

Synthesis of the propargyl Boc amino acid substituents (Boc-AA-Pro)

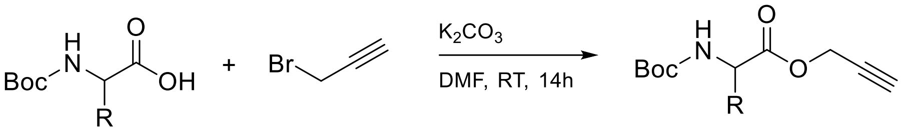

1a: R=CH_3
2a: R=H

Scheme SI 1: General reaction scheme of the synthesis of the propargyl boc amino acids.

First a Boc protected amino acid (AA) (20.0 g, 1.0 equiv.) was dissolved in 250 ml DMF and dried K_2CO_3 (1.15 equiv.) was added. The suspension was then cooled to 0 °C and propargyl bromide (80 wt% solution in toluene) (1.1 equiv.) was added dropwise. After complete addition, the reaction was run at RT for further 14 h. Next the mixture was filtered, and the solvent removed under reduced pressure. The remaining residue was redissolved in EtOAc and washed with brine thrice. Finally, the organic phase was dried with $MgSO_4$ and the solvent removed under reduced pressure, yielding a brownish solid (yield 91 %).

Alanine **1a** (R=CH_3): ^{1}H-NMR (300 MHz, $CDCl_3$, δ / ppm): 5.13 (s, 1H, NH), 4.70-4.58 (m, 2H, -CH_2), 4.24 (s, 1H, CH-CH_3), 2.44 (t (*J*=2.5 Hz), 1H, CH), 1.35 (s, 9H, CH_3), 1.31 (d (*J*=7.2 Hz), 3H, CH-CH_3).

Glycine **2a** (R=H): ^{1}H-NMR (300 MHz, $CDCl_3$, δ / ppm): 4.92 (s, 1H, NH), 4.68 (d (*J*=2.5 Hz), 2H, -CH_2), 3.90 (d (*J*=5.6 Hz), 2H, CH_2), 2.43 (t (*J*=2.5 Hz), 1H, CH), 1.39 (s, 9H, CH_3).

Synthesis of the propargyl amino acid substituents

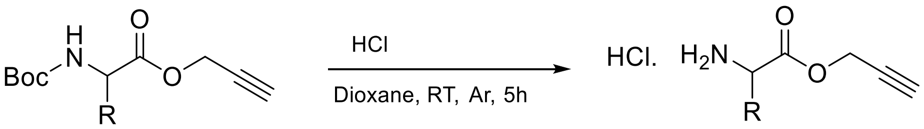

1b: R=CH_3
2b: R=H

Scheme SI 2: General reaction scheme of the synthesis of the AA-Pro substituents.

The Boc protected amino acid derivative (20.4 g, 1.0 equiv.) was dissolved in 30 ml dioxane and an argon atmosphere was established. Then 4M HCl in dioxane (2.15 equiv.) was added to the solution and reacted for 5 h at RT. Next, the formed precipitate was filtered off and washed with dry ether and DCM. Finally, the precipitate was dried under vacuum, yielding the product as a fine white powder (yield 94 %).

Alanine **1b** (R=CH_3): ^{1}H-NMR (300 MHz, $DMSO_{d6}$, δ / ppm): 8.75 (s, 3H, NH_3^+), 4.90-4.80 (m, 2H, CH_2), 4.11 (q (*J*=7,2 Hz), 1H, CH_3-CH), 3.70 (t (*J*=1.4 Hz), 1H, CH), 1.44 (d (*J*=7.2 Hz), 3H, CH_3).

Glycine **2b** (R=H): ^{1}H-NMR (300 MHz, $CDCl_3$, δ / ppm): 8.59 (s, 3H, NH_3^+), 4.85 (d (*J*=2.5 Hz), 2H, CH_2), 3.84 (s, 2H, CH_2), 3.69 (t (*J*=2.5 Hz), 1H, CH).

Synthesis of the phosphorodiamidates 1 Ala-APdA and 2 Gly-APdA

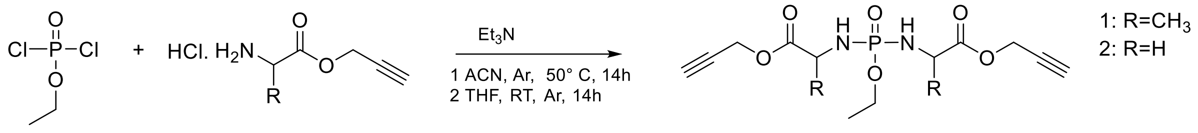

1, 2

Scheme SI 3: Reaction scheme for the synthesis of **1** Ala-APdA and **2** Gly-APdA

The corresponding amino acid substituent (15.0 g, 2.02 equiv.) was dispersed in 250 ml of solvent (**1** ACN, **2** THF) and Et_3N (4.2 equiv.) was added. Then the solution was cooled to 0 °C and ethyl dichlorophosphate (1.0 equiv.) was slowly added under argon atmosphere. After complete addition, the reaction mixture was stirred for 14 h at room temperature (**1**; 50 °C). Next, the precipitate was removed by filtration and the solvent removed under reduced pressure. The remaining residue was redissolved in EtOAc and washed with brine twice. Finally, the organic phase was dried with $MgSO_4$ and evaporated under reduced pressure to yield the product as a viscous liquid (89 %)

1 Ala-APdA (R=CH_3):

^{1}H-NMR (300 MHz, $CDCl_3$, δ / ppm): 4.72-4.60 (m, 4H, CH_2), 3.97 (quint (*J*=7.2 Hz), CH_3-$\underline{CH_2}$-O), 3.92 (s, 2H, NH), 3.35 (q (*J*=9.4 Hz), 2H, CH_3-$\underline{CH}$), 2.48 (t (*J*=2.4 Hz), 2H, CH), 1.35 (dd (J_1=3.7 Hz, J_2=2.8 Hz), 4H, CH_3), 1.21 (t (*J*=7.1 Hz), 3H, $\underline{CH_3}$-CH_2-O).

^{13}C-NMR (75 MHz, $CDCl_3$, δ / ppm): 173.5, 77.2, 75.4. 61.5, 52.6, 49.6, 20.7, 16.2

^{31}P-NMR (121 MHz, $CDCl_3$, δ / ppm): 11.1

ESI-MS: m/z 711.225 $[2M+Na]^+$, 367.106 $[M+Na]^+$, 345.125 $[M+H]^+$

2 Gly-APdA (R=H):

^{1}H-NMR (300 MHz, $CDCl_3$, δ / ppm): 4.68 (d (*J*=2.5 Hz), 4H, CH_2), 4.00 (quint (*J*=7.2 Hz), 2H, CH_3-$\underline{CH_2}$-O), 3.78-3.70 (m, 4H, $\underline{CH_2}$-CH), 3.52 (s, 2H NH), 2.48 (t (*J*=2.5 Hz), 1H, CH), 1.23 (t (*J*=7.1 Hz), 3H, CH_3).

^{13}C-NMR (75 MHz, $CDCl_3$, δ / ppm): 171.8, 77.1, 75.5. 61.7, 52.5, 42.6, 16.2

^{31}P-NMR (121 MHz, $CDCl_3$, δ / ppm): 13.5

ESI-MS: m/z 655.158 $[2M+Na]^+$, 339,075 $[M+Na]^+$, 317.094 $[M+H]^+$

Synthesis of the phoshorodiamidate monomer 3 PdA

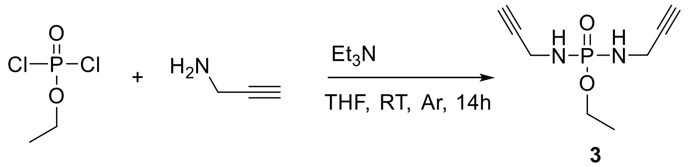

3

Scheme SI 4: Reaction scheme for the synthesis of **3** PdA.

Propargyl amine (8.45 ml, 132 mmol, 2.15 equiv.) was dissolved in 100 ml dry THF and Et_3N (20.1 ml, 145 mmol, 2.4 equiv.) was added. Then the solution was cooled to 0 °C and ethyl dichlorophosphate (10.0 g, 61.4 mmol, 1.0 equiv.) was slowly added under argon atmosphere. After complete addition, the reaction mixture was stirred for 14 h at room temperature. The precipitate was removed by filtration and the solvent removed under reduced pressure. The remaining residue was redissolved in

EtOAc and washed with brine twice. Finally the organic phase was dried with $MgSO_4$ and evaporated under reduced pressure to yield the product as soft brown solid (11.2 g, 56.0 mmol, 92%)

^{1}H-NMR (300 MHz, $CDCl_3$, δ / ppm): 4.07 (quint (*J*=5.8 Hz), 2H, CH_3-$\underline{CH_2}$-O), 3.76-3.69 (m, 2H, CH_2), 3.03 (s, 2H, NH), 2.24 (t (*J*=2.5 Hz), 2H, CH), 1.31 (t (*J*=7.1 Hz), 3H, CH_3).

^{13}C-NMR (75 MHz, $CDCl_3$, δ / ppm): 81.8, 71.1, 61.5, 30.5, 16.2

^{31}P-NMR (121 MHz, $CDCl_3$, δ / ppm): 13.7

ESI-MS: m/z 423.136 $[2M+Na]^+$, 223.062 $[M+Na]^+$, 201.079 $[M+H]^+$

Photopolymerization

Representative procedure for the thiol-ene/yne bulk polymerization of monomers

Monomer 3 PdA (377 mg, 1.9 mmol), trimethylolpropane tris(3-mercaptopropionate) (TMPMP) (1.0 g, 2.5 mmol) and TPO-L (21 mg, 1.5 % wt.) were mixed homogeneously. Then the mixture was cured in the UV reactor at 365 nm for 1.5 - 2 h yielding a crosslinked (thermoset like) resin.

Degradation of monomers

The degradation was monitored at pH values of 7.4 and 3.0 at 37 °C. 30 mg of each monomer was dissolved in 0.9 ml buffer solution (pH 7.4: 1M HEPES, pH 3.0 1M citric acid) and 0.1 ml D_2O and transferred in NMR tubes. The samples were then incubated at 37 °C and ^{31}P NMR were recorded regularly. The degradation progress was determined by using the integrals of monomer and evolving phosphate ester.

Mass loss studies

The degradation of the bulk material was examined by mass loss studies. Cured sample discs (12 mm * 2 mm) were first soaked in EtOAc to extract any unreacted monomers and then dried to record the m_0 value. Then the sample discs were immersed in buffer solutions and incubated at 37 °C (not agitated). In regular time intervals the samples were removed, dried (to constant weight) and their weight was recorded (triplicates). From this data the degradation in bulk was calculated.

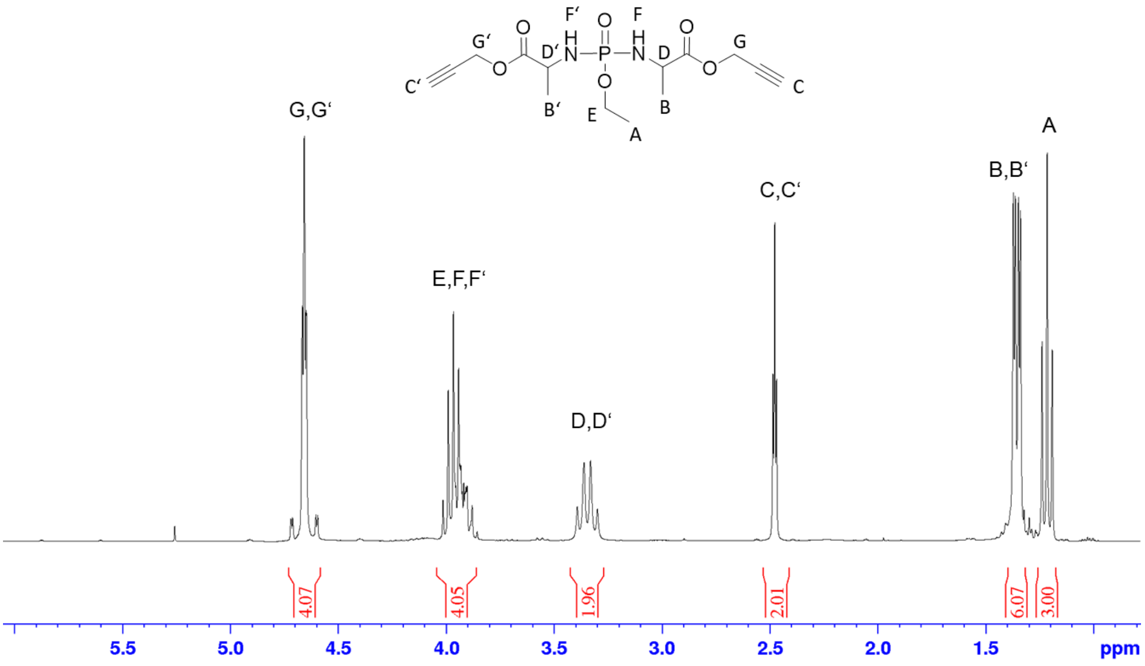

Figure SI-1: Proton NMR of **1** Ala-APdA with integrals and proton assignment (300 MHz, $CDCl_3$).

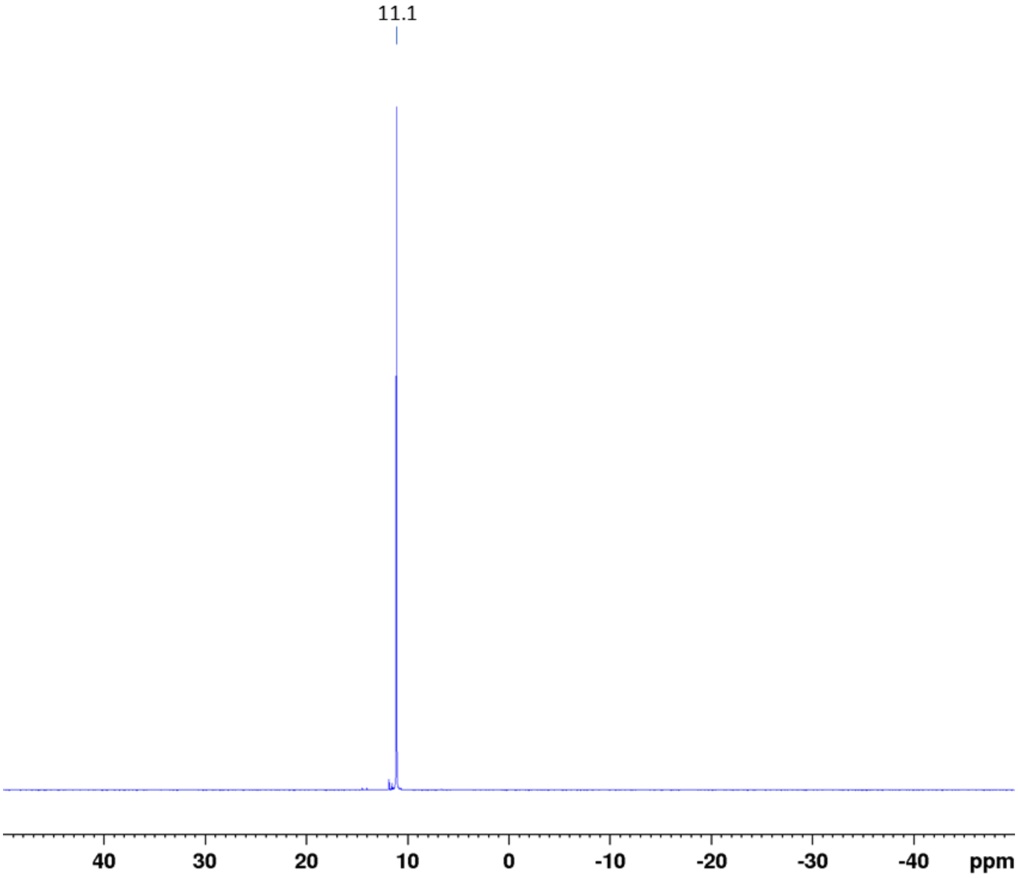

Figure SI-2: ^{31}P NMR of **1** Ala-APdA (121 MHz, $CDCl_3$).

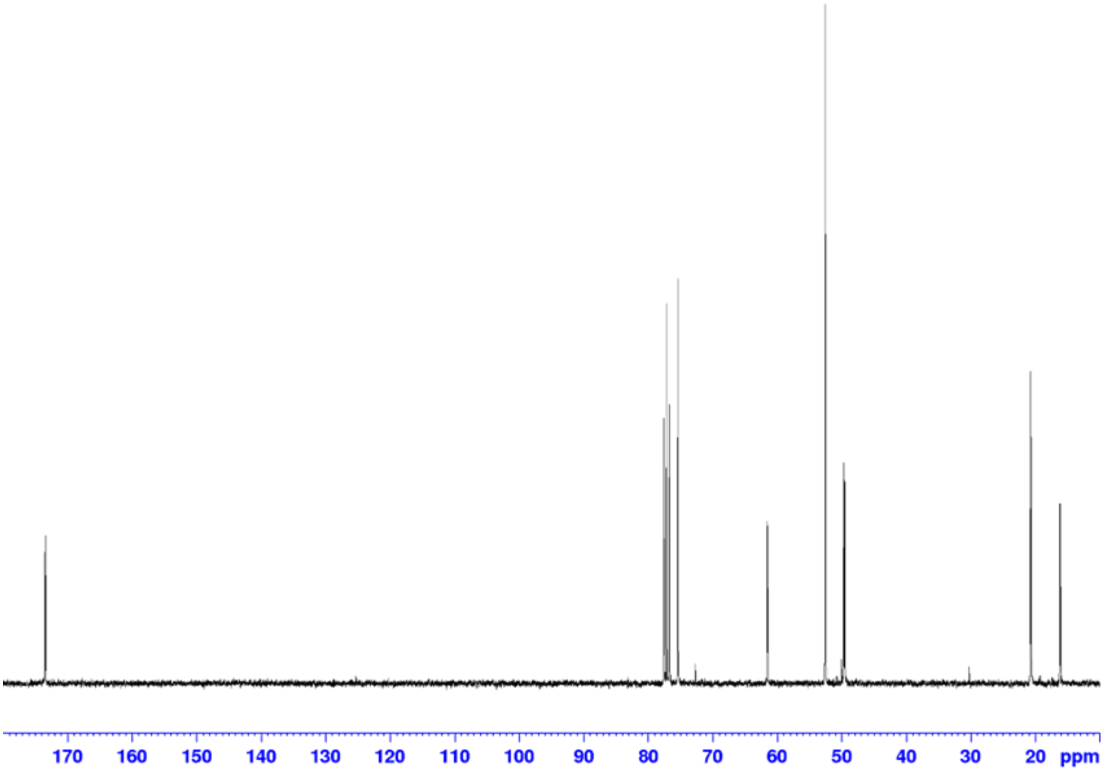

Figure SI-3: ^{13}C NMR of **1** Ala APdA (75 MHz, $CDCl_3$).

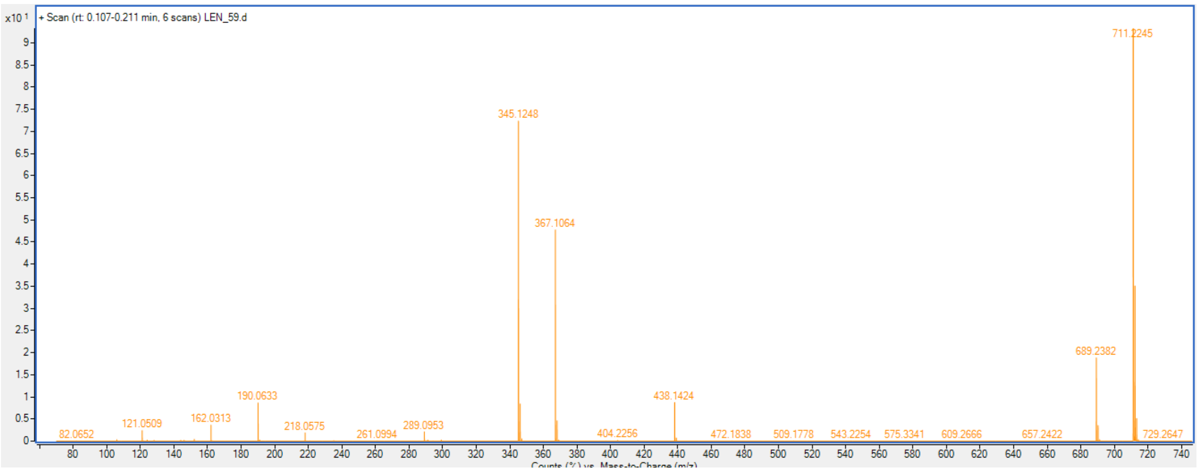

Figure SI-4: ESI-MS spectrum of **1** Ala-APdA; m/z 711.225 [2M+Na]+, 699.238 [2M+H]+, 367.106 [M+Na]+, 345.125 [M+H]+

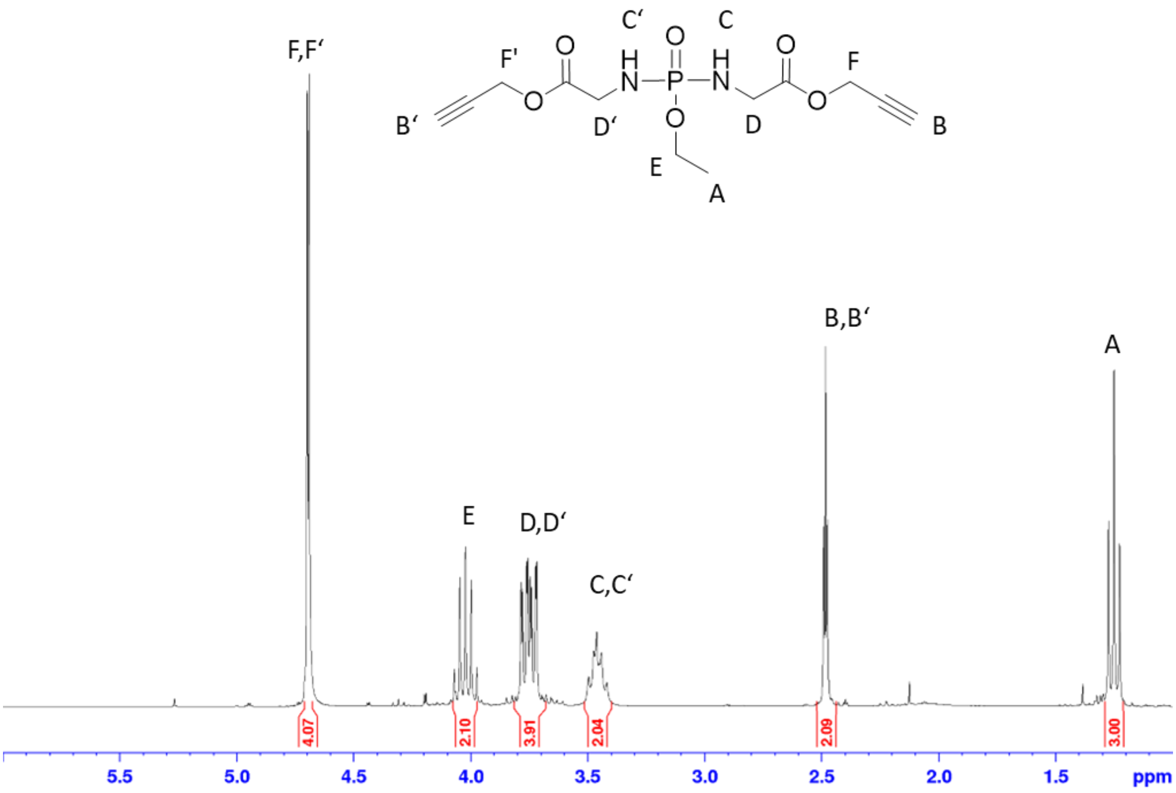

Figure SI-5: Proton NMR spectrum of **2** Gly-APdA with integrals and proton assignment (300 MHz, $CDCl_3$).

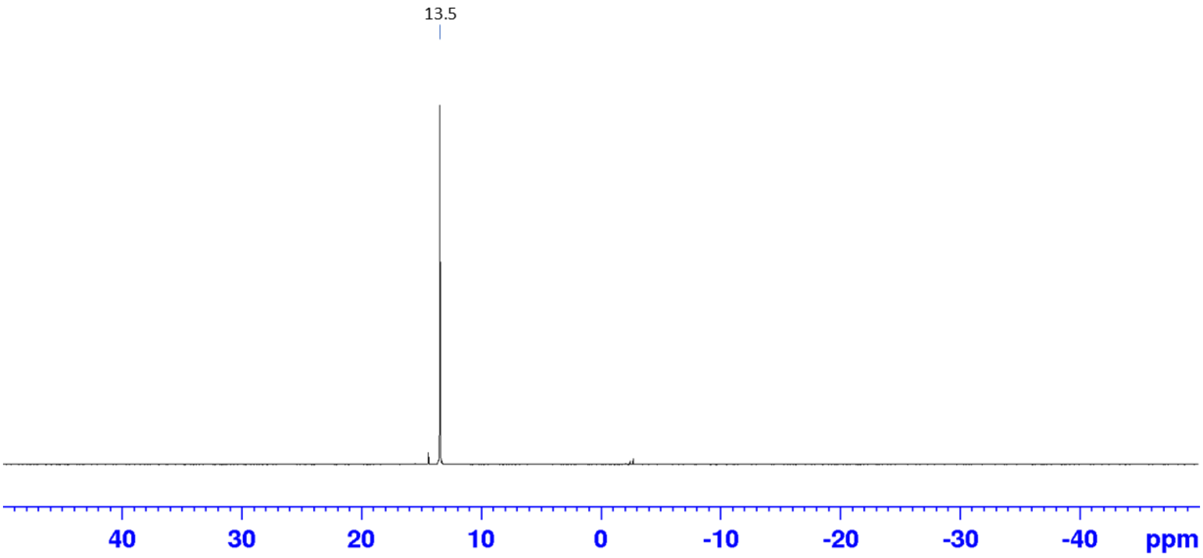

Figure SI-6: ^{31}P NMR of **2** Gly-APdA (121 MHz, $CDCl_3$).

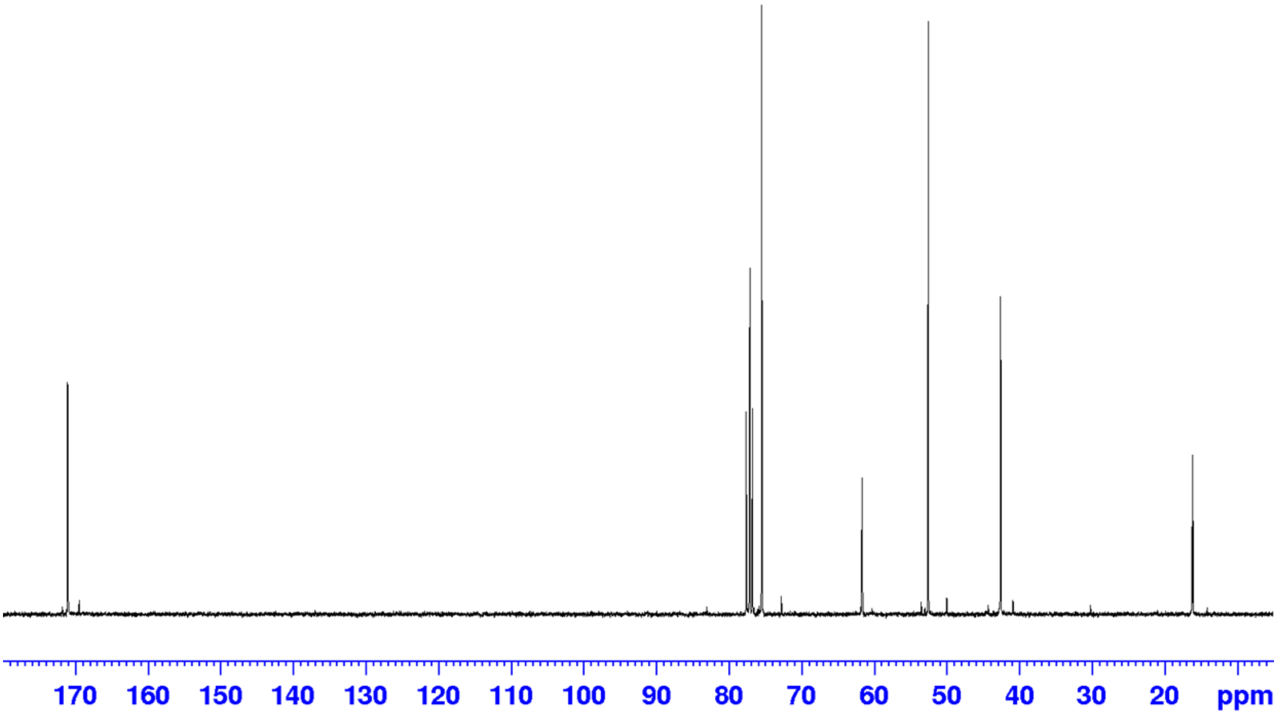

Figure SI-7: ^{13}C NMR of **2** Gly-APdA (75 MHz, $CDCl_3$)

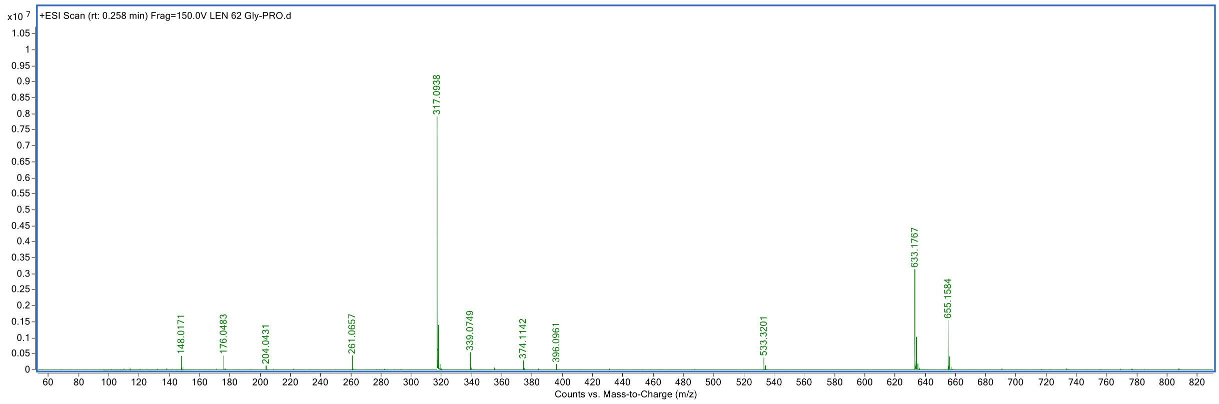

Figure SI-8: ESI-MS spectrum of **2** Gly-APdA; m/z 655.158 [2M+Na]+, 633.177 [2M+H]+, 339.075 [M+Na]+, 317.094 [M+H]+

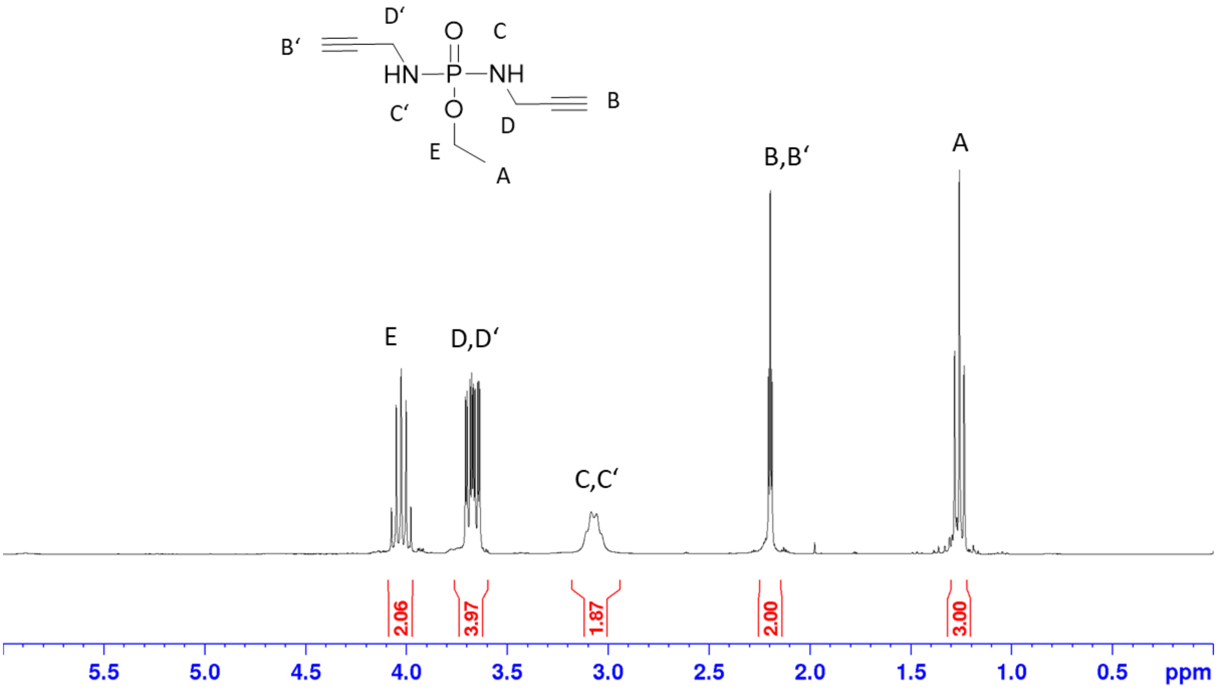

Figure SI-9: Proton NMR of 3 PdA with integrals and proton assignment (300 MHz, $CDCl_3$).

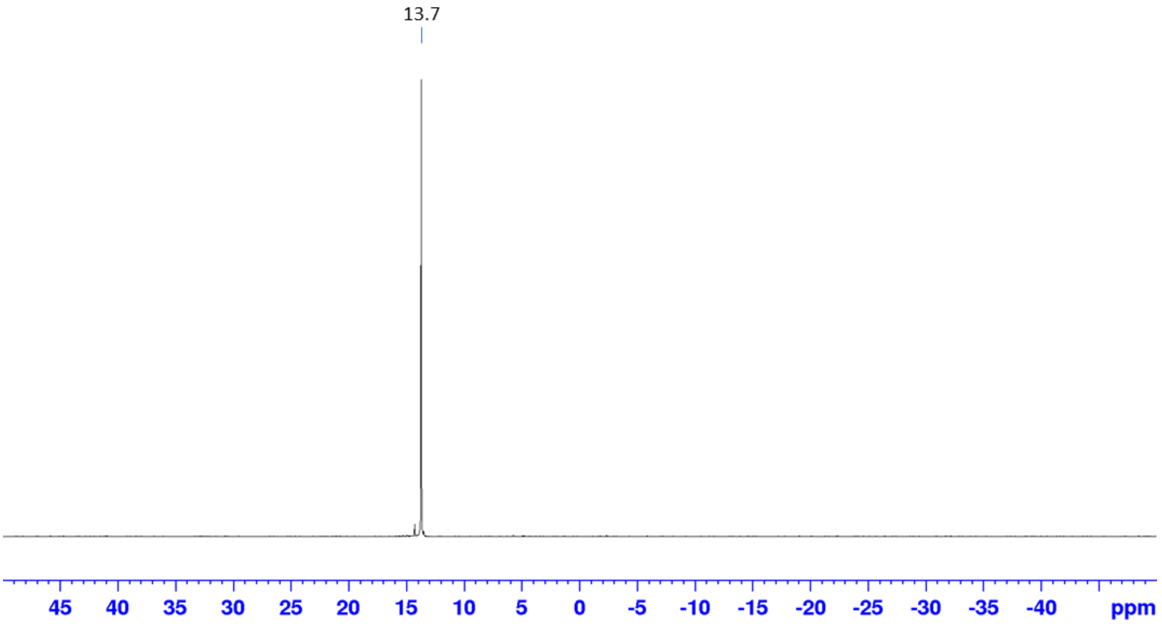

Figure SI-10: ^{31}P NMR of **3** PdA (121 MHz, $CDCl_3$).

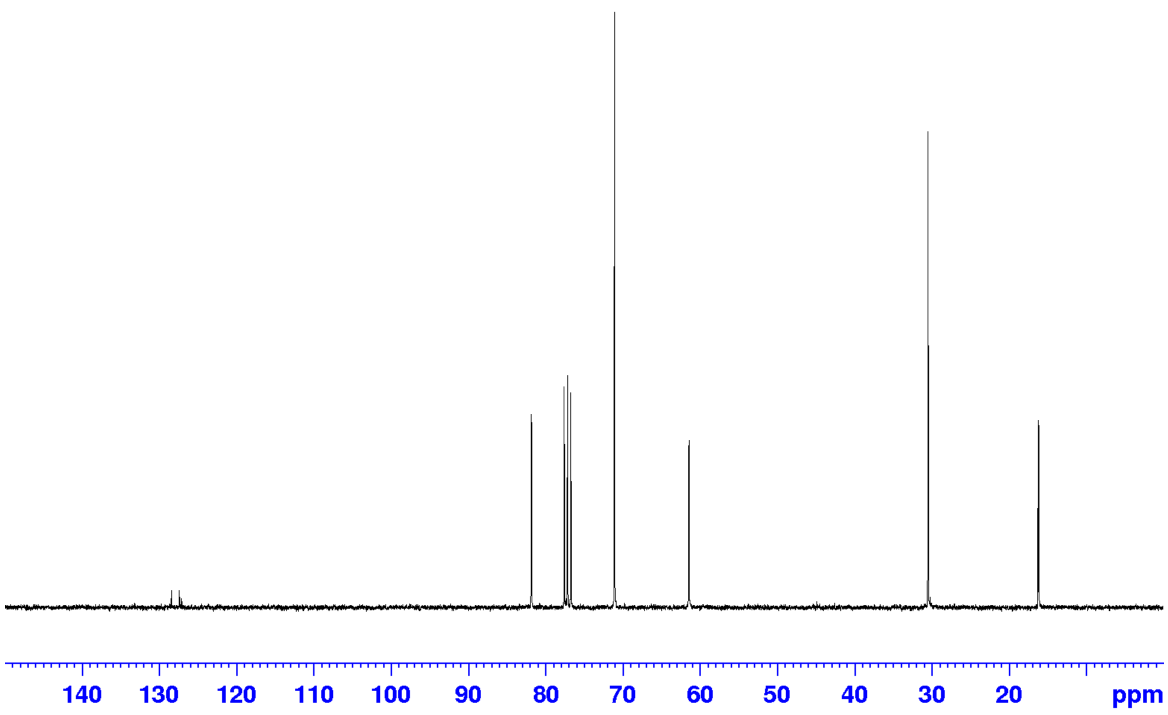

Figure SI-11: ^{13}C NMR of **3** PdA (75 MHz, $CDCl_3$).

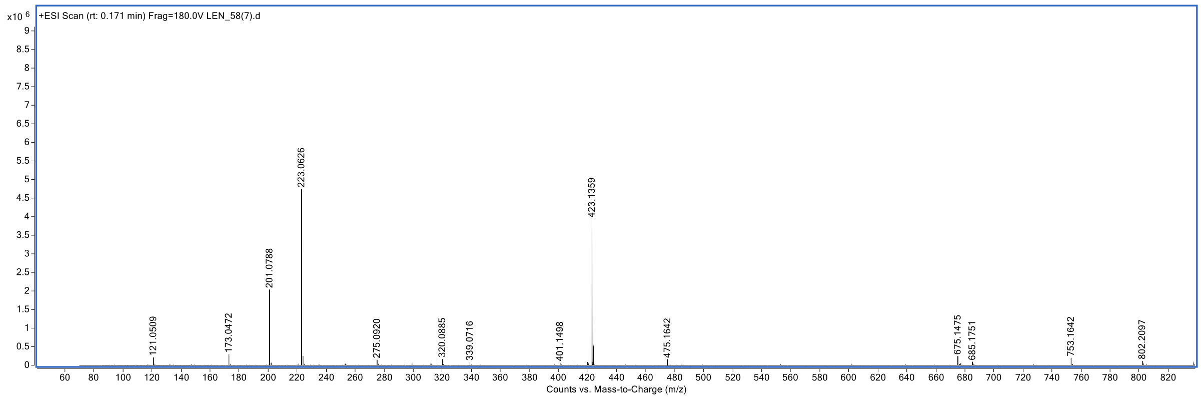

Figure SI-12: ESI-MS spectrum of **3** PdA; m/z: 423.136 $[2M+Na]^+$, 223.063 $[M+Na]^+$, 201.079 $[M+H]^+$

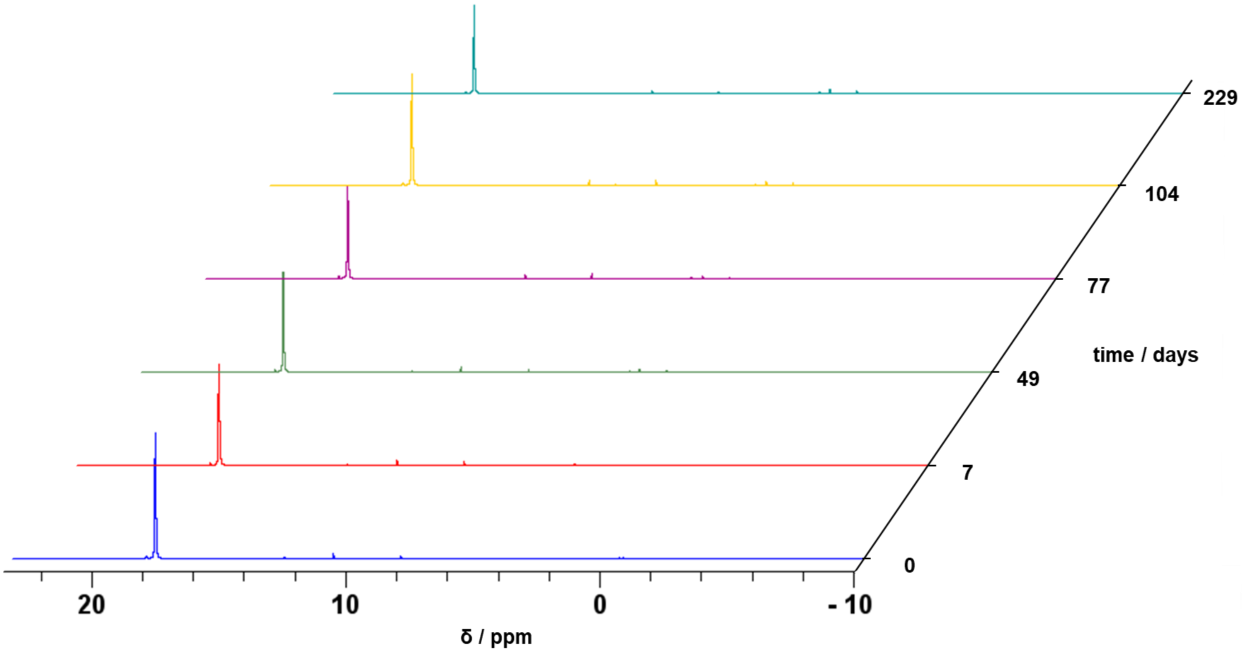

Figure SI-13: Degradation of PdA at pH 7.4 at 37° C monitored by ^{31}P-NMR. No degradation was observed during the whole period until day 229, as indicated by the absence of any phosphate peak thus showing the stability of PdA against hydrolysis at pH 7.4

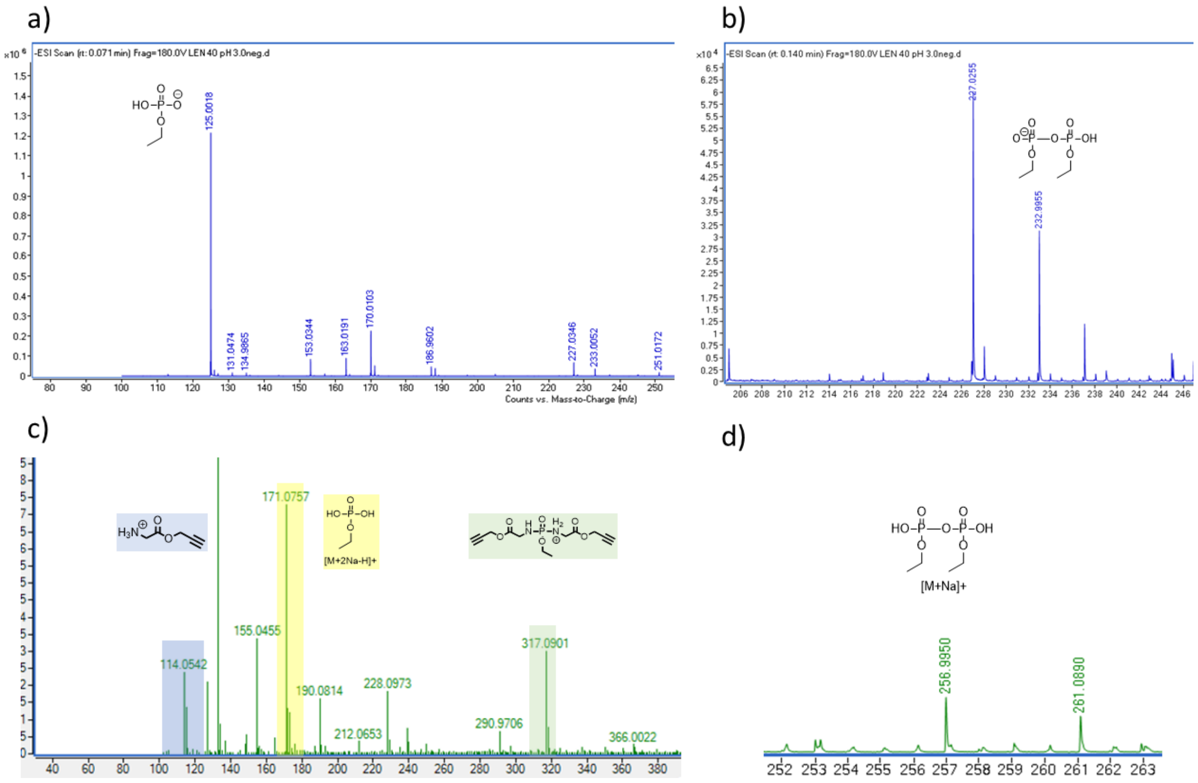

Figure SI-14: MS spectra of the degradation products of **2** Gly-APdA at pH 3.0. a) and b) negative ion mode showing the phosphate ester as final degradation product m/z 125.002 [M-H]- (a) and the dimer

m/z 232.996 [M-H]- (b). Interestingly, the mono substituted Gly-APdA as an intermediate degradation product was not observed at pH 3.0, suggesting a rapid further hydrolysis once one of the amino acid substituents has been hydrolyzed. c) and d) positive ion mode; c) the spectrum shows the amino acid substituent m/z 114.054 [M+H]+, the sodium adduct of the phosphate ester m/z 171.076 [M+2Na-H]+ and the monomer m/z 317.090 [M+H]+. Spectra d) shows the sodium adduct of the dimer phosphate ester m/z 256.995 [M+Na]+.

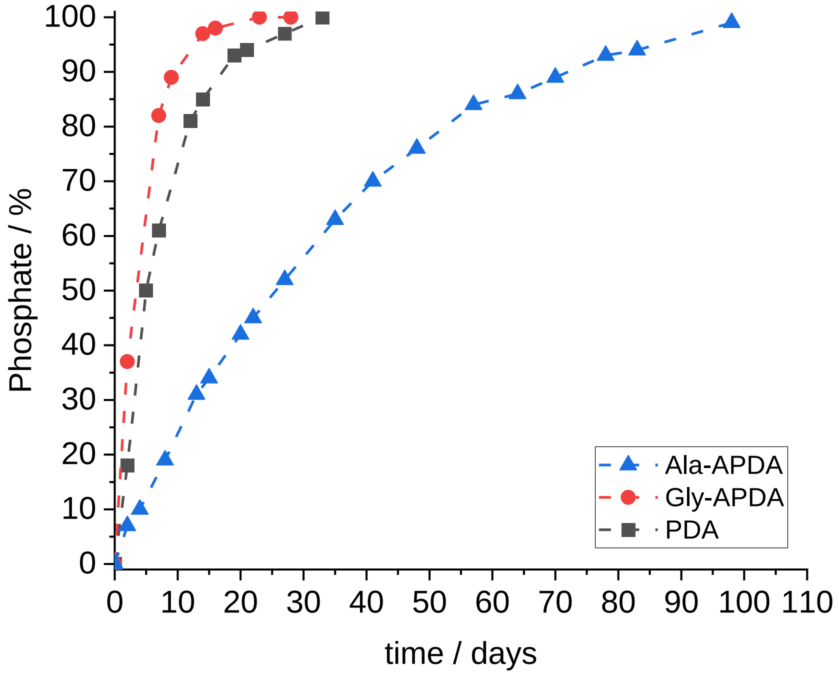

Figure SI-15: Hydrolysis of monomers 1-3 at pH 3.0 and 37 °C, determined by phosphate formation in ^{31}P NMR spectroscopy. The hydrolysis is accelerated at pH 3.0 for all monomers. Ala-APdA shows the slowest rate, which can be attributed to the shielding effect of the methyl group.

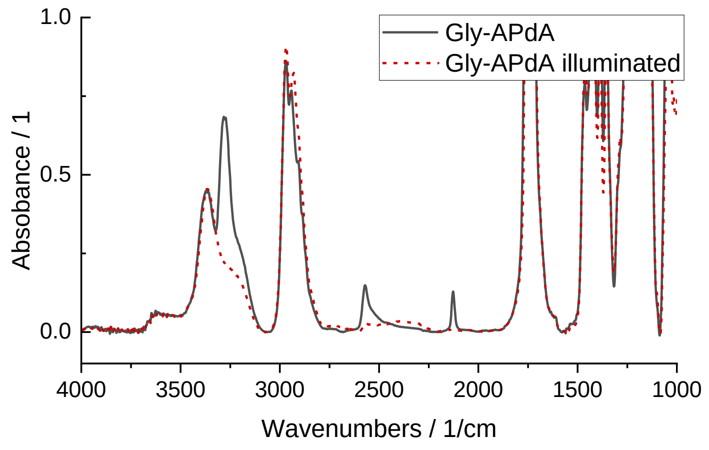

Figure SI-16: FTIR of Gly-APdA with TMPMP before and after illumination with UV light. Photopolymerization was achieved by activation with TPO-L and can be followed by the decrease in alkyne (3280 cm^{-1} and 2130 cm^{-1}) as well as the thiol (2570 cm^{-1}) signals.

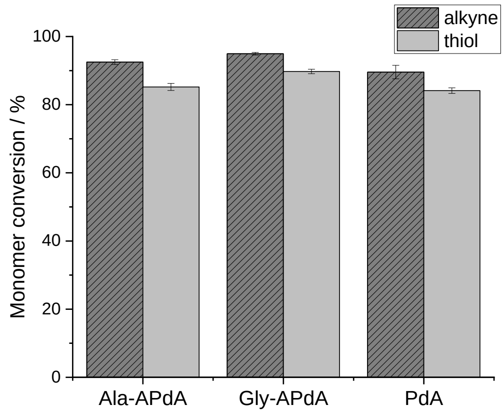

Figure SI-17: Final monomer conversion as measured by RT-FTIR. The disparity between the final monomer conversions of alkyne and thiol groups can be attributed to the formation of vinyl sulfide as a reaction intermediate, which can undergo homopolymerization without consuming a second thiol group.[2,3]

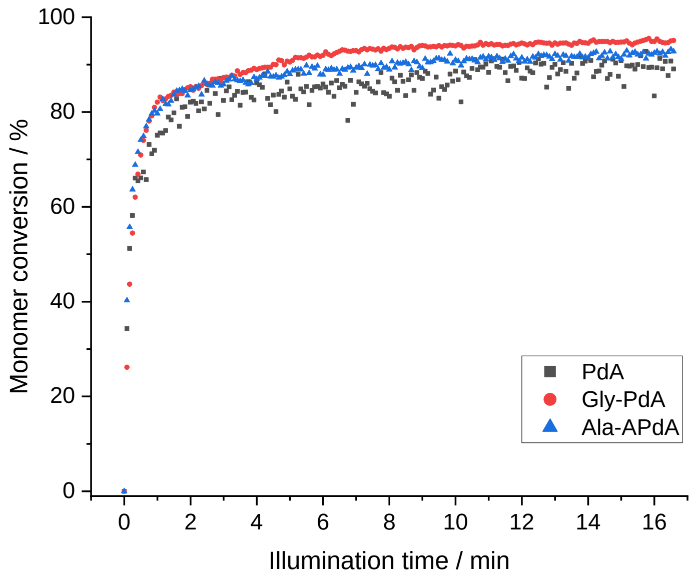

Figure SI-18: Monomer conversion during photopolymerization (250-650 nm; 75 mW cm^{-2}) was monitored over the illumination time and measured by RT-FTIR.

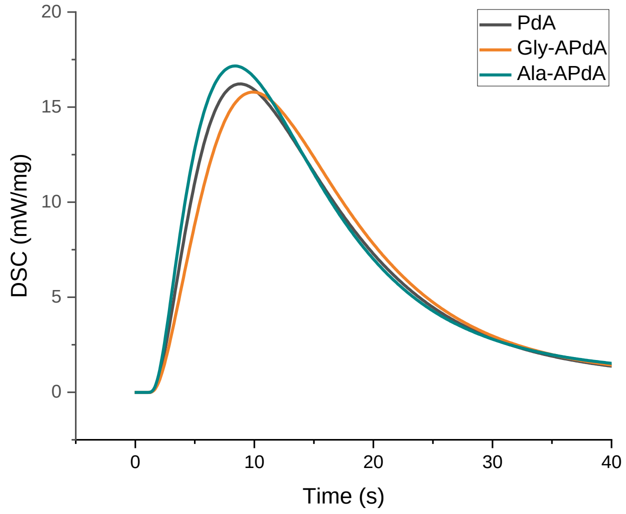

Figure SI-19. Heatflow during photopolymerization (300-500 nm; 292 mW cm^{-2}) investigated by photo-DSC.

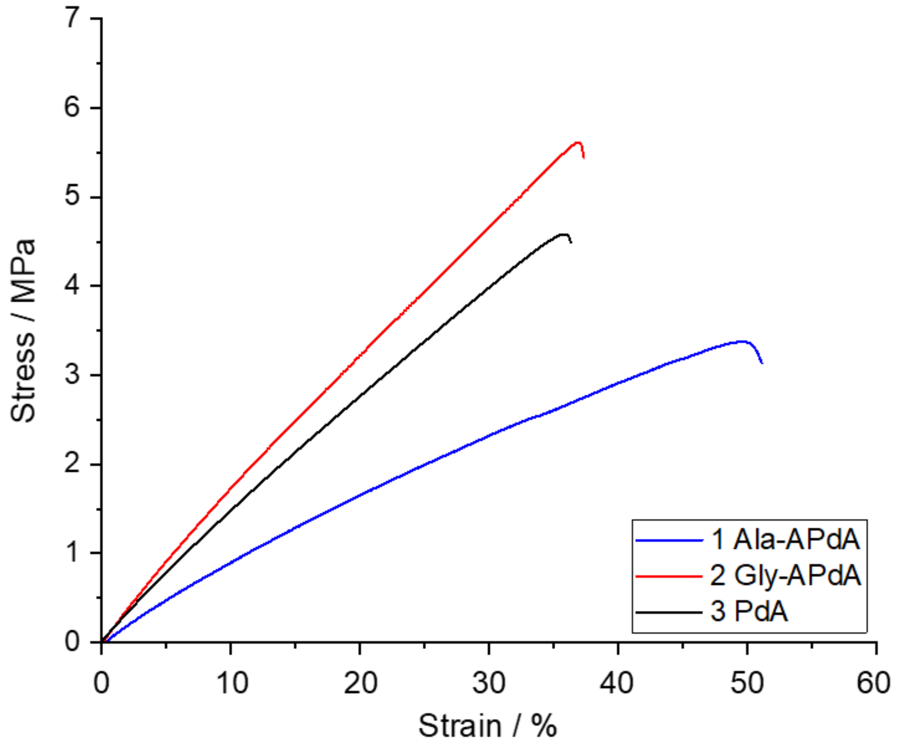

Figure SI-20: Tensile test of 1 Ala-APdA, 2 Gly-APdA and 3 PdA at a rate of 0.1 mm s^{-1} using dog-bone shaped specimen until failure. The samples exhibit near linear elastic behaviour and brittle break, with yield strains in similar ranges of 45-55 %.

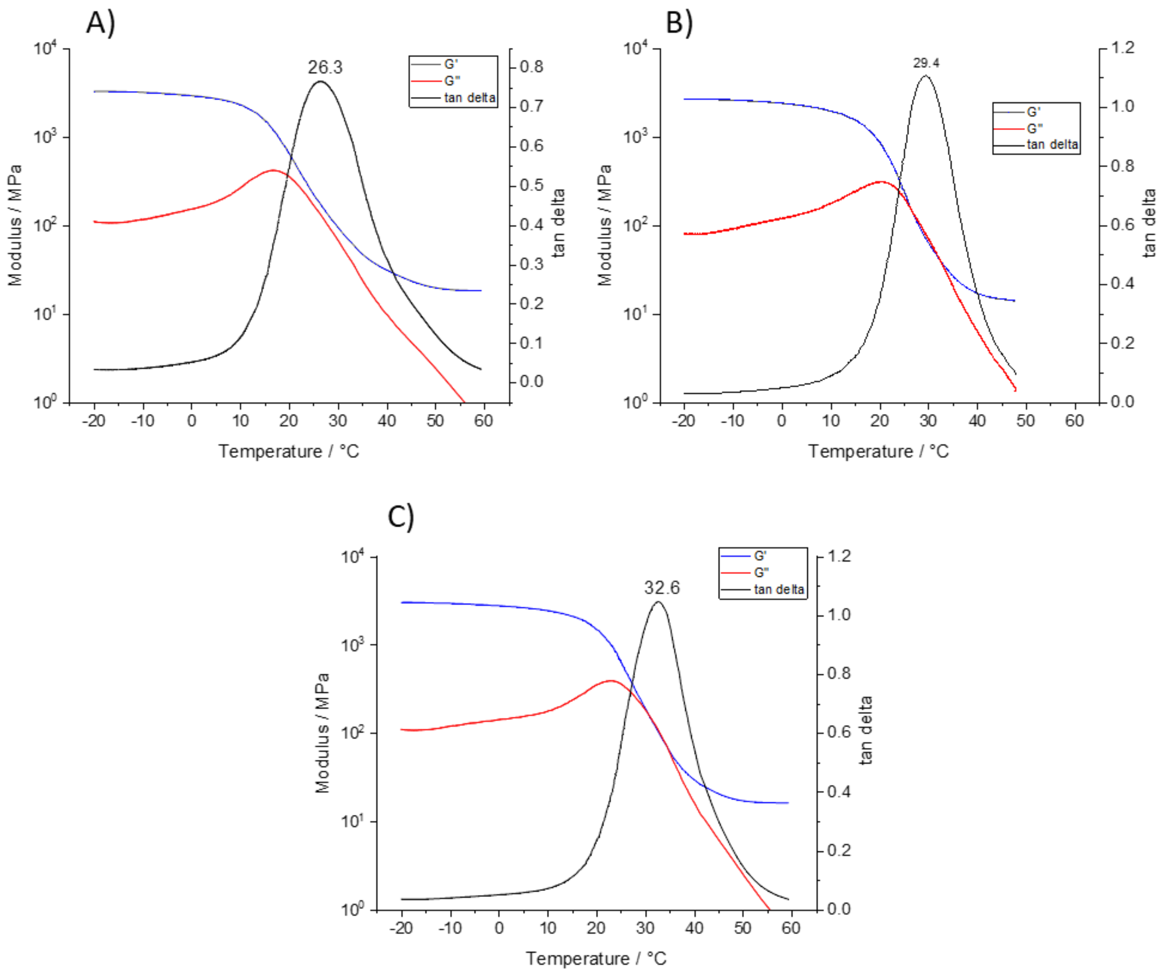

Figure SI-21: DMA measurements of A) PdA, B) Gly-APdA and C) Ala-APdA, with the T_g marked as the maximum of the tan delta curve. Compared to PdA, Gly-APdA and Ala-APdA show a higher T_g which can be attributed to the incorporated amino acid spacer which leads to higher steric hinderance and lower chain mobility. This effect is further increased by the additional methyl group of Ala-APdA, which therefore has the highest observed T_g.

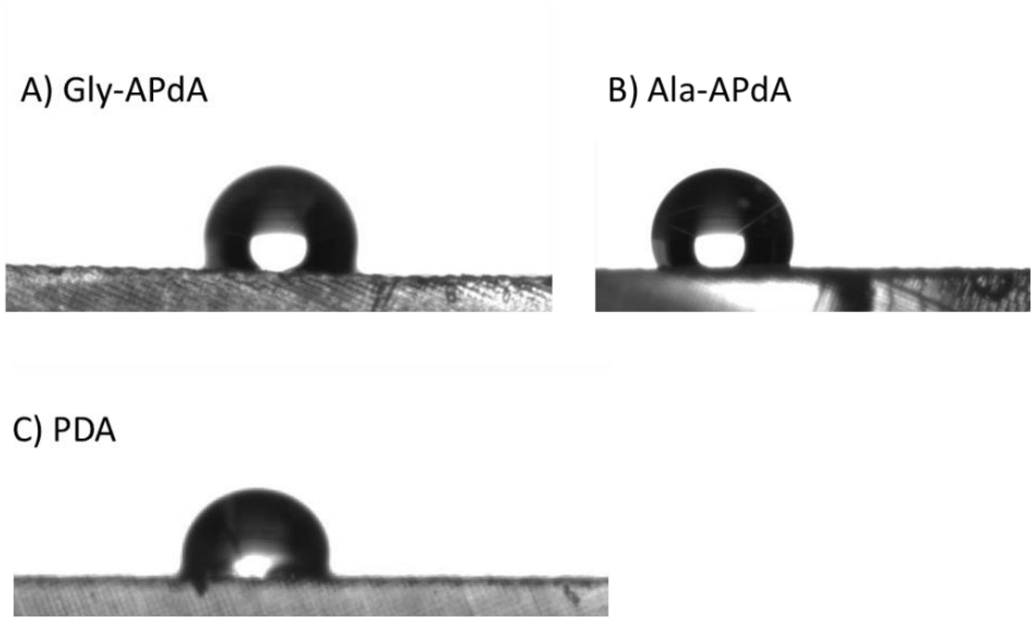

Figure SI-22: Pictures of the contact angle measurements of the different formulations. As can be seen the materials hydrophobic properties prevail: A) Gly-APdA: 98 °, B) Ala-APda 98 ° and C) PDA 73 °.

References

(1) Buchroithner, B.; Hartmann, D.; Mayr, S.; Oh, Y. J.; Sivun, D.; Karner, A.; Buchegger, B.; Griesser, T.; Hinterdorfer, P.; Klar, T. A.; Jacak, J. 3D multiphoton lithography using biocompatible polymers with specific mechanical properties. *Nanoscale Advances* **2020**, *2* (6), 2422–2428. DOI: 10.1039/D0NA00154F.

(2) Fairbanks, B. D.; Scott, T. F.; Kloxin, C. J.; Anseth, K. S.; Bowman, C. N. Thiol-Yne Photopolymerizations: Novel Mechanism, Kinetics, and Step-Growth Formation of Highly Cross-Linked Networks. *Macromolecules* **2009**, *42* (1), 211–217. DOI: 10.1021/ma801903w. Published Online: Oct. 12, 2008.

(3) Hennen, D.; Hartmann, D.; Rieger, P. H.; Oesterreicher, A.; Wiener, J.; Arbeiter, F.; Feuchter, M.; Fröhlich, E.; Pichelmayer, M.; Schlögl, S.; Griesser, T. Exploiting the Carbon and Oxa Michael Addition Reaction for the Synthesis of Yne Monomers: Towards the Conversion of Acrylates to Biocompatible Building Blocks. *ChemPhotoChem* **2020**, *4* (7), 476–480. DOI: 10.1002/cptc.201900199.
